# Clinical outcomes of patients with *mut*-type methylmalonic acidemia identified through expanded newborn screening in China

**DOI:** 10.1186/s40246-024-00646-0

**Published:** 2024-07-29

**Authors:** Shiying Ling, Shengnan Wu, Ruixue Shuai, Yue Yu, Wenjuan Qiu, Haiyan Wei, Chiju Yang, Peng Xu, Hui Zou, Jizhen Feng, Tingting Niu, Haili Hu, Huiwen Zhang, Lili Liang, Yu Wang, Ting Chen, Feng Xu, Xuefan Gu, Lianshu Han

**Affiliations:** 1 Department of Pediatric Endocrinology/Genetics, Shanghai Institute for Pediatric Research, Xinhua Hospital, School of Medicine, Shanghai Jiao Tong University, Shanghai, China; 2 Department of Endocrinology and Metabolism, Henan Key Laboratory of Children’s Genetics and Metabolic Diseases, Henan Children’s Hospital, Children’s Hospital Affiliated to Zhengzhou University, Zhengzhou Children’s Hospital, Zhengzhou, 450018 China; 3 Center of Neonatal Disease Screening, Jining Maternal and Child Health Care Hospital, Jining, China; 4 Center of Neonatal Disease Screening, Jinan Maternal and Child Health Care Hospital, Jinan, China; 5 Center of Neonatal Disease Screening, Shijiazhuang Maternal and Child Health Care Hospital, Shijiazhuang, China; 6 Center of Neonatal Disease Screening, Shandong Maternal and Child Health Care Hospital, Jinan, China; 7 Center of Neonatal Disease Screening, Hefei Maternal and Child Health Care Hospital, Hefei, China

**Keywords:** Methylmalonic acidemia, *MMUT* gene, Long-term outcome, Newborn screening

## Abstract

**Background:**

Isolated methylmalonic acidemia, an autosomal recessive disorder of propionate metabolism, is usually caused by mutations in the methylmalonyl-CoA mutase gene (mut-type). Because no universal consensus was made on whether *mut*-type methylmalonic acidemia should be included in newborn screening (NBS), we aimed to compare the outcome of this disorder detected by NBS with that detected clinically and investigate the influence of NBS on the disease course.

**Design & methods:**

In this study, 168 patients with *mut*-type methylmalonic acidemia diagnosed by NBS were compared to 210 patients diagnosed after disease onset while NBS was not performed. Clinical data of these patients from 7 metabolic centers in China were analyzed retrospectively, including initial manifestations, biochemical metabolites, the responsiveness of vitamin B12 therapy, and gene variation, to explore different factors on the long-term outcome.

**Results:**

By comparison of the clinically-diagnosed patients, NBS-detected patients showed younger age at diagnosis, less incidence of disease onset, better responsiveness of vitamin B12, younger age at start of treatment, lower levels of biochemical features before and after treatment, and better long-term prognosis (*P* < 0.01). Onset of disease, blood C3/C2 ratio and unresponsiveness of vitamin B12 were more positively associated with poor outcomes of patients whether identified by NBS. Moreover, the factors above as well as older age at start of treatment were positively associated with mortality.

**Conclusions:**

This research highly demonstrated NBS could prevent major disease-related events and allow an earlier treatment initiation. As a key prognostic factor, NBS is beneficial for improving the overall survival of infants with *mut*-type methylmalonic acidemia.

**Supplementary Information:**

The online version contains supplementary material available at 10.1186/s40246-024-00646-0.

## Introduction

Isolated methylmalonic acidemia (OMIM # 251,000), an autosomal recessive disorder, is characterized by increased levels of propionylcarnitine (C3) and methylmalonic acid in the blood, urine, and other body fluids that result from the failure to convert the methylmalonyl-coenzyme (CoA) into succinyl-CoA during propionate metabolism in the mitochondrial matrix, without hyperhomocysteinemia [1]. This disorder is usually caused by a complete (mut^0^ enzymatic subtype) or partial (mut^−^ enzymatic subtype) deficiency of methylmalonyl-CoA mutates, most encoded by the *MMUT* gene [2, 3]. Isolated methylmalonic acidemia occurs in 1:110 000 live births in all regions worldwide. In China, isolated methylmalonic acidemia accounts for typically 30% of all types, and the *MMUT* gene deficiency accounts for 93.5% of the isolated methylmalonic acidemia [4–6].

The clinical spectrum of *mut*-type methylmalonic acidemia is heterogeneous [7]. Affected patients could present the first clinical symptoms during the neonatal period, even immediately after birth, but commonly develop within the first year of life [8], which are characterized by variable and non-specific clinical signs such as poor feeding, vomiting, drowsiness, coma, hyperammonemia, and metabolic crises [9, 10]. Moreover, patients who survive the initial acute metabolic acidosis usually get into chronic progression and suffer from anemia, renal dysfunction, epilepsy, developmental delay, psychological behavior abnormalities, and other long-term complications [9, 10].

Nowadays, the introduction of tandem mass spectrometry (MS/MS) to newborn screening for inborn errors of metabolism has been a great success. In China, MS/MS-based NBS was first applied in Shanghai. A total of 116,000 newborns underwent the NBS from 2003 to 2007, only 3 methylmalonic acidemia patients were identified suggesting an incidence of 1:40000 [11]. However, subsequent studies have shown the prevalence of this disease varied considerably in different regions of China, especially between the north and the south [6, 12]. In recent years, *mut*-type methylmalonic acidemia is usually discovered through NBS, undoubtfully this could increase the chance of an early diagnosis of patients [13], especially in late-onset cases [14]. However, there is limited information available on the long-term outcome of screened individuals [8, 14, 15]. Therefore, we compared the natural history and prognosis of patients diagnosed through NBS and those diagnosed on clinical bases to illustrate the benefit of NBS programs for individuals with *mut*-type methylmalonic acidemia and to investigate the effect of variable factors on the screened outcome.

## Materials and methods

### Study design

A total of 378 patients diagnosed and treated at multiple hospitals in China from January 2003 to December 2021 were enrolled, and we divided patients into two cohorts. Patients identified by MS/MS-based NBS (*n* = 168) comprised the first cohort, defined as the NBS-detected cohort. The other cohort included patients diagnosed by onset of first symptoms (*n* = 210), for which NBS was not performed. Then patients were divided into two groups, with the normal outcome group and the poor outcome group suffering from different degrees of physical and mental impairment, such as movement disorders, intellectual impairment, developmental delay, renal or cardiac complications evaluated by the clinical endocrinologist, and death. The details of standardized criteria for evaluation have been described as previously published [16]. For the Gesell Developmental Scale test, the result of DQ ≥ 86 indicates normal development; DQ 75 ~ 85 indicates possible neurocognitive impairment and/or movement disorders; DQ *≤* 74 indicates varying degrees of neurocognitive impairment and/or movement disorders. In total, 238 patients with poor outcomes in the whole cohort and 73 patients with poor outcomes in the NBS-detected cohort were respectively analyzed by univariate logistic regression as well as Cox regression. Factors that included the age at disease onset and start of treatment, the onset of symptoms, the practice of NBS, unresponsiveness of vitamin B12, nucleotide variants, and biochemical markers before and after treatment were analyzed. This study was approved by the Ethics Committee of Xinhua Hospital Affiliated to Shanghai Jiao Tong University School of Medicine (Approval number: XHEC-D-2022-062). Informed consent was obtained from all patients or their legal guardians for being included in the study.

### Newborn screening

From January 2003 to December 2021, almost 60% of newborns in China were enrolled for MS/MS-based NBS program. According to the recommended time frame [17], dried blood spots (DBSs) from newborns aged 3 to 7 days were taken by heel puncture on filter paper (Waterman Company, Columbus, OH). Each DBS information card will record the infant’s gestational age and birth weight. Then DBS was air-dried at room temperature and delivered to the laboratory of Newborn screening centers nationwide within 5 days. Samples were tested with MS/MS (API 4000, American Bio-System Inc). Cut-off values were set by determining > 10,000 normal infants (0.5th percentile − 99.5th percentile) and were adjusted at intervals with increased sample data and clinical data to reduce false positives and false negatives. Cases with results above the cutoff value for C3 and the C3/ acetylcarnitine (C2) ratio were judged as positives. Screened positive samples were recalled for repeated MS/MS tests. If a repeat sample demonstrated a persistent increase both in the C3 level and the C3/C2 ratio, then further confirmatory tests, including urine organic acid and genetic analysis, were conducted. Individuals excluded from having the disease through repeated MS/MS tests as well as other confirmatory tests were regarded as false positives.

### Metabolites analysis

Sample preparation for MS/MS was performed using both derivatized (January 2003 to June 2022) and non-derivatized (June 2021 to June 2022) methods. According to the derivatized pre-treatment method [18], DBSs were extracted with methanol containing isotope-labeled amino acids and acylcarnitines as internal standards. Then the n-butyl alcohol-hydrochloric acid was used for derivatization. The levels of acylcarnitines in blood, including C3 and C2 were detected by MS/MS. Then the C3/C2 ratio was calculated. And the reference ranges for C3 and C3/C2 ratio were 0.5-4.0µmol/L, 0.04–0.2, respectively. Second blood spots were requested upon abnormal first-screening result [19]. After that, the following gas chromatography-mass spectrometry (GC-MS) (Shimadzu Limited, QP2010) was used to measure the level of organic acids in urine, which contain methylmalonic acid (MMA) and methylcitric acid (MCA) [20, 21]. Quantification of each organic acid was achieved by calculating the relative peak area of each Q-ion to that of the corresponding internal standard. The normal ranges of MMA and MCA were 0.2-3.6mmol/mol Cr and 0-0.8mmol/mol Cr. Furthermore, all patients had normal levels of plasma total homocysteine, which was assessed by fluorescence polarization immunoassay (normal control < 15 µmol/L) to exclude the cases with methylmalonic acidemia combined with homocysteinemia.

### Mutation analysis of the *MMUT* gene

Genetic analysis was performed by Sanger sequencing or high-throughput next-generation sequencing. The mutation was identified by the normal human *MMUT* sequence as a reference (GenBank, NC_000006.12). Then the ClinVar database (https://www.ncbi.nlm.nih.gov/clinvar/), the Human Gene Mutation Database (HGMD, http://www.hgmd.cf.ac.uk/ac/index.php), and the previous literature were used to identify whether the mutations had been reported. The pathogenicity of novel variants was interpreted according to the American College of Medical Genetics and Genomics (ACMG) standards and guidelines [22].

### Diagnosis of *mut*-type methylmalonic acidemia

Diagnosis of mut-type methylmalonic acidemia is based on clinical manifestations, abnormal levels of diagnostic metabolites, which include C3, C3/C2 ratio, MMA and MCA, and genetic results of at least one pathogenic variant in *MMUT* gene.

### Evaluation of the responsiveness to vitamin B12

According to the guidelines and following the proposed protocol by Fowler et al., the responsiveness to vitamin B12 should be determined in all affected individuals [2, 10, 23]. Therefore, in our study, affected individuals should be given 1 mg or 5 mg of vitamin B12 intramuscularly every day for five days, followed by an assessment of the production of related metabolites to judge whether the treatments were effective. For example, an improvement in clinical symptoms and a significant (> 50%) reduction in the blood C3/C2 ratio and urinary methylmalonic acid level after the vitamin B12 loading test are considered indicative of complete responsiveness. If the blood C3/C2 ratio and urinary methylmalonic acid levels decrease by < 50% but > 30%, it was deemed as partly vitamin B12 responsive [24]. Otherwise, the injection of vitamin B12 was considered non-responsive if a decreased value in the blood C3/C2 ratio and urinary methylmalonic acid were < 30%.

### Treatment

For these patients, treatment varies with the difference in vitamin B12 responsiveness and is adjusted depending on personal conditions [10]. In the acute decompensation, all patients were intravenously administered levocarnitine (50–100 mg/kg, 1–2 times a day). After the symptoms were relieved, levocarnitine was given orally 100–300 mg/ (kg·d) (based on the clinical response and carnitine levels). According to the guideline, for vitamin B12 responsive patients, the long-term therapy mainly involved cobalamin intramuscular injections (5-10 mg, 2–3 times a week) to maintain the levels of C3, C3/C2 ratio, MMA and MCA in the desired range.

For the partly vitamin B12 responsive patients, the treatment should be combined with cobalamin intramuscular injections and a special formula supplementation. For vitamin B12 unresponsive patients, the main treatment was a special diet and nutritional interventions [23]. The natural protein intake was adjusted to 0.8–1.5 g/(kg·d) depending on the individual. At the same time, a specially formulated nutritional powder without isoleucine, valine, threonine, and methionine was given to supplement protein 0.2–1.0 g/(kg·d) to maintain the total daily protein intake at 1.0–2.5 g/( kg·d) for the needs of growth and development.

### Statistical analysis

The data were analyzed using SPSS 24.0 (IBM, Chicago, Illinois). Descriptive statistics were expressed as the median and the range for continuous variables and as percentages (%) for categorical variables. Baseline clinical, biochemical, and genetic characteristics were compared between patients whether detected by NBS and patients with different prognoses using a Student independent t-tests, χ2/Fisher exact test, or Wilcoxon rank-sum test as appropriate. The univariable logistic regression model was used to identify the associated factors of poor outcomes. Cox regression was performed to identify potentially associated mortality factors. A value of *P* < 0.05 was considered statistically significant.

## Results

### Patients population

Among 168 patients in the NBS-detected cohort, 21 cases (12.5%) died and 8 cases (4.8%) were lost to follow-up. While, in 210 cases identified after disease onset, 69 cases (32.9%) died and 12 cases (5.7%) were lost to follow-up. A comparison of demographic variables between those identified by NBS with or without poor outcomes was described in Table 1 and a comparison between patients whether diagnosed by NBS was presented in Table 2.

**Table 1:** Comparison of baseline demographic, clinical, and biochemical characteristics between patients with good and poor outcomes in the NBS-detected cohort

Characteristics	Normal outcome (*n* = 87)	Poor outcome (*n* = 73)	*P* value
Age at diagnosis (months), median (range)	1 (21 days-3.5 months)	1.44 (1–6 months)	0.02
Age during follow-up (years), median (range)	5.52 (1.75–18.21 years)	4.78 (2.32–13.98 years)	0.14
Gender, n (%)			0.88
Male	44(50.57%)	37(48.68%)	
Female	43(49.43%)	36(51.32%)	
Disease onset, n (%)	14 (16.09%)	73 (100%)	0.00
Age at onset of first symptoms (days), median (range)	90 (3 days-1.9 years)	3 (1 day-2.33 years)	0.01
Initial symptoms, n (%)			
Milk refusal	2 (2.3%)	37 (50.68%)	0.00
Vomiting	10 (11.49%)	31 (42.49%)	0.00
Drowsiness	7 (8.05%)	33 (45.21%)	0.00
Seizures	4 (4.6%)	12 (16.44%)	0.02
Coma	2 (2.3%)	18 (24.66%)	0.00
Dyskinesia	3 (3.45%)	18 (24.66%)	0.00
Progressively developmental delay	1 (1.15%)	13 (17.81%)	0.00
Vitamin B12 responsiveness, n (%)	75 (86.21%)	17 (23.29%)	0.00
Age at treatment of vitamin B12 (months), median (range)	1 (21 days-4.7 months)	1.32 (21 days − 87.3 months)	0.02
Biochemical features at NBS, median (range)			
Blood C3 level (µmol/L)	6.3(2.11–40.42)	9.91 (3.25–54.85)	0.00
Blood C3/C2 ratio	0.45 (0.12–1.72)	0.70 (0.26–4.3)	0.00
Urinary MMA (mmol/mol Cr)	104.3 (4.62–921)	272.9 (14.85–5915)	0.00
Urinary MCA (mmol/mol Cr)	3.48 (0.30-39.32)	12.30 (0.8-90.88)	0.00
Biochemical features during follow-up			
Blood C3 level (µmol/L)	5.00 (0.83–55.36)	24.44 (2.22–68.3)	0.00
Blood C3/C2 ratio	0.22 (0.03–1.4)	0.89 (0.06–1.67)	0.00
Urinary MMA (mmol/mol Cr)	19.75 (0-928.7)	352.2 (0-2685)	0.00
Urinary MCA(mmol/mol Cr)	0.96 (0–53)	4.5 (0-60.23)	0.00
Nucleotide variant, n (%)			
c.729_730insTT	17 (19.54%)	17 (23.29%)	0.70
c.1663G > A	33 (37.93%)	2 (2.74%)	0.00
c.1106G > A	6 (6.9%)	18 (24.66%)	0.00
c.323G > A	8 (9.2%)	7 (9.59%)	1.00
c.914T > C	3 (3.45%)	9 (12.33%)	0.07
c.424 A > G	4 (4.6%)	5 (6.85%)	0.74
c.755dupA	5 (5.75%)	3 (4.11%)	0.73
c.599T > C	6 (6.9%)	8 (10.96%)	0.29
c.1677-1G > A	3 (3.45%)	4 (5.48%)	0.71
c.1280G > A	1 (1.16%)	5 (6.85%)	0.10
c.2080 C > T	4 (4.6%)	2 (2.74%)	0.69
c.1630_1631GG > TA	4 (4.6%)	1 (1.37%)	0.37
others	59 (67.82%)	58 (76.32%)	0.30

Normal reference range of blood C3: 0.5-4.0µmol/L; Normal reference range of blood C3/C2 ratio: 0.04–0.2; Normal reference range of urinary MMA: 0.2-3.6mmol/mol Cr; Normal reference range of urinary MCA:0-0.8mmol/mol Cr

**Table 2:** Comparison of baseline demographic, clinical, and biochemical characteristics between patients identified by NBS or clinical manifestations

Characteristics	NBS-detected cohort (*n* = 168)	Clinically-diagnosed cohort (*n* = 210)	*P* value
Age at diagnosis (months), median (range)	1 (21 days-6 months)	2.5 (1 month-8 years)	0.00
Age during follow-up (years), median (range)	5.03 (1.75–18.21 years)	8.5 (2.49–35.06 years)	0.00
Gender, n (%)			0.03
Male	85 (50.6%)	131 (62.38%)	
Female	83 (49.4%)	79 (37.62%)	
Disease onset, n (%)	91 (54.17%)	210 (100%)	0.00
Initial symptoms, n (%)			
Milk refusal	41 (24.4%)	119 (56.67%)	0.00
Vomiting	43 (25.6%)	121 (57.62%)	0.00
Drowsiness	42 (25%)	107 (50.95%)	0.00
Seizures	16 (9.52%)	44 (20.95%)	0.00
Coma	21 (12.5%)	44 (20.95%)	0.03
Dyskinesia	22 (13.1%)	63 (30%)	0.00
Progressively developmental delay	15 (8.93%)	59 (28.1%)	0.00
Vitamin B12 responsiveness, n (%)	92 (54.76%)	70 (33.33%)	0.01
Age at treatment of vitamin B12 (months), median (range)	1 (21 days-7.33 years)	6 (30 days-17 years)	0.00
Biochemical features at baseline, median (range)			
Blood C3 level (µmol/L)	7.88(2.11–54.85)	11.49(2.19–81.13)	0.00
Blood C3/C2 ratio	0.58(0.12–4.3)	0.72(0.19–4.51)	0.01
Urinary MMA (mmol/mol Cr)	160.9(4.62–5915)	366(6.2-15038)	0.00
Urinary MCA (mmol/mol Cr)	6.29(0.3-90.88)	9.05(0.3-250.8)	0.02
Biochemical features during follow-up			
Blood C3 level (µmol/L)	7.79(0.83–68.3)	19.61(0.95–84.22)	0.00
Blood C3/C2 ratio	0.38(0.03–1.67)	0.78(0.06-2)	0.00
Urinary MMA (mmol/mol Cr)	67.56(0-2685)	242(0-3632)	0.01
Urinary MCA (mmol/mol Cr)	1.87(0-103.6)	3.8(0-89.35)	0.21
Outcome at follow-up, n (%)			
Normal outcome	87 (51.79%)	33 (15.71%)	0.00
Poor outcome	73 (43.45%)	165 (78.57%)	

Normal reference range of blood C3: 0.5-4.0µmol/L; Normal reference range of blood C3/C2 ratio: 0.04–0.2; Normal reference range of urinary MMA: 0.2-3.6mmol/mol Cr; Normal reference range of urinary MCA:0-0.8mmol/mol Cr

### Age at onset, diagnosis, and the start of vitamin B12 therapy

In patients identified by NBS, the diagnosis was made between 21 days and 6 months after birth, with a median age of 1 month. In the clinically-diagnosed cohort, the median age at diagnosis was 2.5 months, ranging more widely from 1 month to 8 years. As for the age at the treatment of vitamin B12, the median ages in the two cohorts were 1 month and 6 months respectively. Undoubtedly, NBS led to significantly earlier diagnosis as well as earlier treatment (both *P* < 0.01). However, in 54.2% of patients detected by NBS, the first symptoms were already noted between 1 day and 28 months of life. This implies that a proportion of them had already exhibited their first symptoms before receiving NBS results. Indeed, 45 of 168 (26.8%) patients who were identified by NBS had shown clinical symptoms during the newborn period. In the NBS-detected cohort, the age at first symptoms of patients with normal outcome was 90 days (range 3 days to 1.9 years), while 3 days (range 1 day to 2.3 years) in the poor outcome group. Noteworthy, the age at onset was younger in the poor outcome group than in the normal outcome group among patients identified by NBS (*P* = 0.01). Moreover, the age of both at diagnosis and therapy of vitamin B12 were consistently lower in the normal outcome group (both *P* = 0.02).

### Initial manifestation

As shown in Table 2, among patients identified by NBS, 91 of 168 (54.2%) developed symptoms eventually. While 77 of 168 (45.8%) have not presented any symptoms so far. A considerable number of patients exhibited clinical manifestations during the first 30 days of life, thus being classified as early-onset patients. Fortunately, 14 cases had disease onset beyond day 30, which all showed normal psychomotor and cognitive development at our last visit. The most common phenotype of both early-onset and late-onset children was metabolic acidosis, such as aversion to eating and vomiting, which were found in 77.9% (60/77) and 57.1% (8/14) of both groups, respectively. Neurological symptoms ranked second, including drowsiness, coma, and seizures that occurred in 52 patients (67.5%) in the early-onset ones and 7 cases (50%) in the late-onset ones, suggesting clinical symptoms were more severe in patients with early-onset. In the clinical picture of all patients identified either by NBS or disease onset, all showed significant differences. In both cohorts, vomiting and drowsiness were the most prominent symptoms. Furthermore, a comparison of the occurrence of different kinds of clinical features was made between individuals with or without normal physical and neurocognitive development in the NBS-detected cohort. Consistently, the incidence of variable initial symptoms was significantly higher in the poor outcome group (*P* < 0.05), indicating the onset of disease might have a negative effect on the long-term prognosis.

### Biochemical features

According to Table 1, before treatment, the poor outcome group showed higher levels of C3, C3/C2 ratio, MMA and MCA than the normal group (all *P* < 0.01). Additionally, these metabolites after treatment varied in individuals with different outcomes, in which all of them were much lower in the normal outcome group than those in the poor outcome group, with significant statistical differences (all *P* < 0.01). In the NBS-detected cohort, 5 cases showed C3/C2 ratios within the normal range, but their blood C3 levels were above the cutoff, thus being screened positive. These 5 cases all showed normal development. Meanwhile, only 9 newborns had blood C3 levels under 4 µmol/L, whereas the C3/C2 ratios were over 0.2. Among them, only one patient whose C3/C2 ratio was 1.15 showed developmental delay, while the blood C3/C2 ratios of the other eight children were a little higher than the cutoff. At the same time, all 8 patients remained asymptomatic and showed normal outcomes during the follow-up (Supplementary Table 1), which points out that the C3/C2 ratios might be helpful in optimizing the sensitivity and specificity for diagnosis. Therefore, more emphasis should be placed on the analysis of the acylcarnitine inter-ratio during MS/MS-based NBS. In addition, there were remarkable differences in biochemical data that included the blood C3, C3/C2 ratio, urinary MMA, and MCA between patients identified by NBS and clinical symptoms (Table 2), which implied a potential relationship between biochemical indicators and prognosis.

### Gene analysis

In the NBS-detected cohort, *MMUT* mutations were observed on both alleles in 161 of 168 cases (95.8%), whereas in 7 cases (4.2%) a single mutation on one allele was detected. Sequence analysis identified 107 mutations in the *MMUT* gene, including 64 missense mutations, 19 nonsense mutations, 3 duplications, 11 deletions, 4 insertions, and 6 splice-site mutations (Supplementary Table 1). Between groups with or without good outcomes in the NBS-detected cohorts or cohorts whether patients were identified by NBS or clinical symptoms, only the variant of c.1663G > A(p.A555T) strongly differed (*P* < 0.01). For patients who had a favorable long-term outcome, the variant c.1663G > A(p.A555T) seemed more common (37.9% versus 2.6%). In analogy, the variant of c.1663G > A(p.A555T) in *MMUT* was increasingly observed in the NBS-detected cohort than in the clinically-diagnosed cohort. Additionally, we noted that patients carrying the variant of c.753 + 3 A > G had milder clinical phenotype, lower levels of biochemical metabolites and even merely receiving vitamin B12 orally could have a better prognosis. But this mutation in the *MMUT* gene is relatively rare, only 3 cases were found in our study. Therefore, through NBS receiving disease-specific therapy and preventing disease progression, patients carrying the above-mentioned variants in our cohort seemed to have a better prognosis.

### Factors affecting poor outcomes

**Fig. 1:**
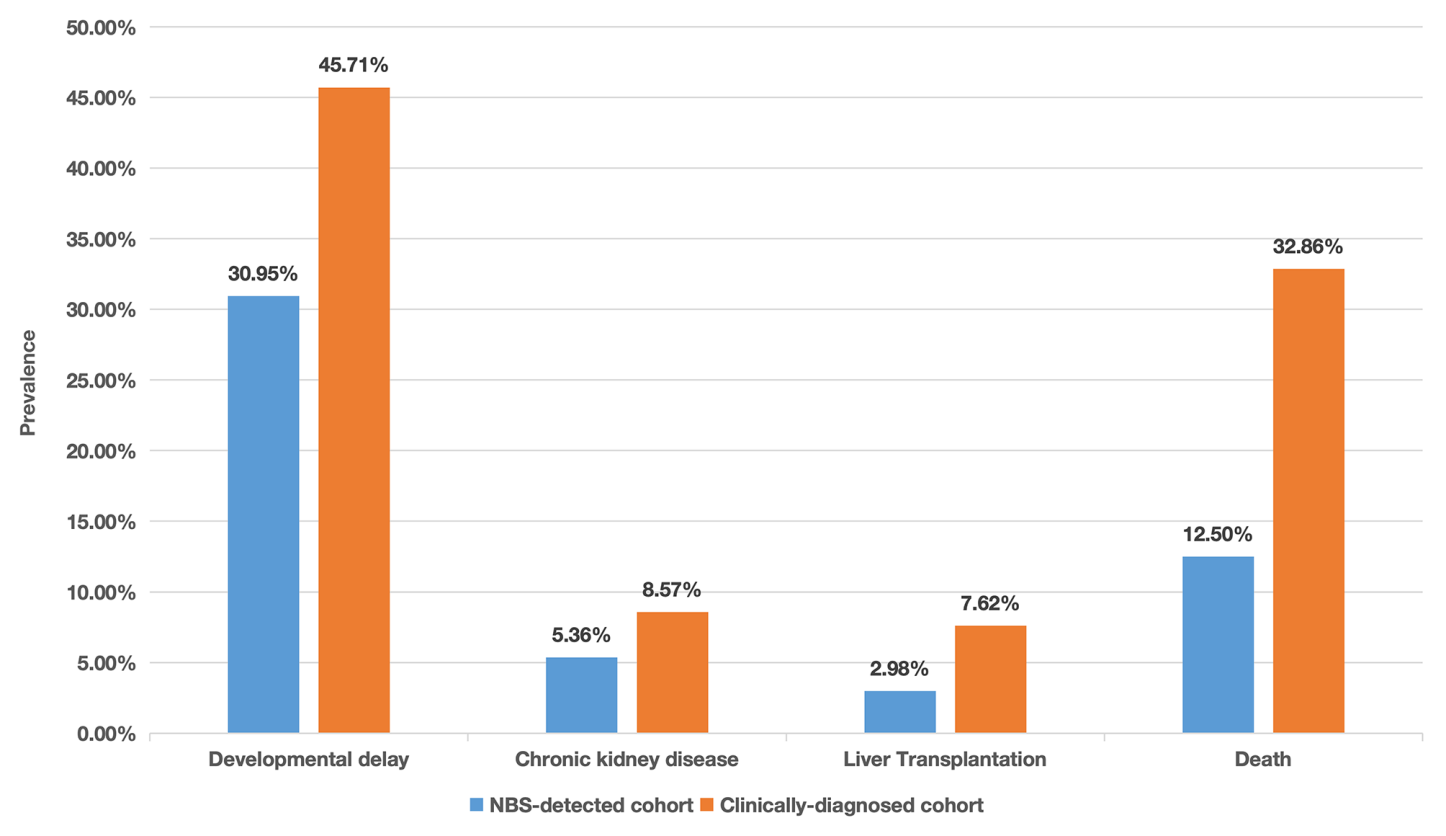
Detailed poor outcomes of patients in the NBS-detected cohort and clinically-diagnosed cohort

In the NBS-detected cohort, 73 patients (43.5%) had poor outcomes, in which 21 patients died of recurrent and severe metabolic crises. In the clinically-diagnosed cohort, unfortunately, 165 cases (78.6%) had poor outcomes, including 96 cases showing different degrees of developmental delay and 69 deceased cases. In addition, the mortality rate was much lower (12.5% versus 32.7%) in the NBS-detected group compared to the individuals diagnosed after disease onset (*P* < 0.01). Among them, the age at death of about two-thirds of patients was before 1 year of age, and most patients die of respiratory failure and renal failure due to metabolic acidosis. Besides, 27 patients showed varying degrees of renal impairment, including 9 patients diagnosed through NBS and 18 diagnosed by disease onset first. For cognitive evaluation, 47 children received the test of Gesell Developmental Scale. Consequently, 22 patients had normal development (DQ ≥ 86) of which 12 were in the NBS cohort and 10 were in the clinically-diagnosed cohort (60% versus 37%), six patients had possible neurocognitive deficits and/or motor disabilities (DQ 75–85) including five in the NBS cohort and one in the clinically-diagnosed cohort (25% versus 3.7%), 19 patients had varying degrees of neurocognitive deficits and/or motor disabilities (DQ *≤* 74), including three patients diagnosed through NBS and 16 diagnosed by disease onset first (15% versus 59.3%). A total of 21 patients underwent liver transplantation due to recurrent metabolic instability and ineffective drug control, in which five of them were identified by NBS while the other 16 were identified by the occurrence of metabolic crisis (Fig. 1). After transplantation, only one patient who was also detected by NBS developed healthy, others still showed developmental delay or intellectual impairment.

Both in the univariate analysis for the whole cohort which included NBS-detected group and clinically-diagnosed group and for the subjects with poor outcomes and also underwent NBS, the onset of first symptoms, unresponsiveness of vitamin B12, blood C3 levels and C3/C2 ratios, urinary MMA levels before and after treatment were more likely to predict poor outcomes (Table 3). In contrast, the practice of NBS and the presence of c.1663G > A were associated with more favorable outcomes. Interestingly, when we took NBS into consideration, the odds ratio of disease onset decreased and the unresponsiveness to vitamin B12 increased, which illustrates that NBS could prevent the disease course by allowing treatment started as soon as possible. Moreover, the univariate logistic regression indicated that the younger age at disease onset was negatively associated with prognosis, while the age at the start of treatment was not associated with the risk of poor outcome.

**Table 3:** Univariate analysis of predictors for poor outcome in the whole cohort and NBS-detected cohort

	The whole cohort						NBS-detected cohort	
	OR	95% CI	*P* value			OR	95% CI	*P* value
Age at disease onset	0.51	0.37–0.7	0.00			0.26	0.07–0.93	0.04
Disease onset	29.38	4.24–46.80	0.00			16.35	3.5-46.02	0.00
NBS	0.19	0.12–0.31	0.00					
Biochemical features at baseline								
Blood C3 level (µmol/L)	1.09	1.05–1.14	0.00			1.11	1.05–1.19	0.00
Blood C3/C2 ratio	11.52	4.94–26.89	0.00			11.58	3.77–35.59	0.00
Urinary MMA (mmol/mol Cr)	1.00	1.00–1.00	0.01			1.00	1.00-1.01	0.00
Urinary MCA (mmol/mol Cr)	1.01	1.00-1.02	0.27			1.00	0.99–1.01	0.75
Biochemical features at follow-up								
Blood C3 level (µmol/L)	1.10	1.07–1.13	0.00			1.10	1.06–1.13	0.00
Blood C3/C2 ratio	56.36	21.16-150.13	0.00			49.47	13.40-182.70	0.00
Urinary MMA (mmol/mol Cr)	1.00	1.00–1.00	0.00			1.00	1.00–1.00	0.00
Urinary MCA (mmol/mol Cr)	1.10	1.05–1.16	0.00			1.07	1.02–1.13	0.01
Vitamin B12 unresponsiveness	3.32	2-5.52	0.00			4.74	2.22–10.13	0.00
Age at start of treatment	1.00	0.99–1.01	0.56			1.11	0.92–1.34	0.28
Nucleotide variant								
c.729_730insTT	1.47	0.82–2.62	0.20			1.42	0.62–3.25	0.41
c.1663G > A	0.06	0.02–0.17	0.00			0.07	0.02–0.32	0.00
c.1106G > A	1.71	0.81–3.60	0.16			1.96	0.72–5.40	0.19
c.323G > A	2.02	0.90–4.56	0.09			1.16	0.36–3.80	0.80
c.2080 C > T	0.33	0.10–1.08	0.07			0.22	0.03–1.92	0.17

OR: odds ratio, CI: confidence interval.

**Fig. 2:**
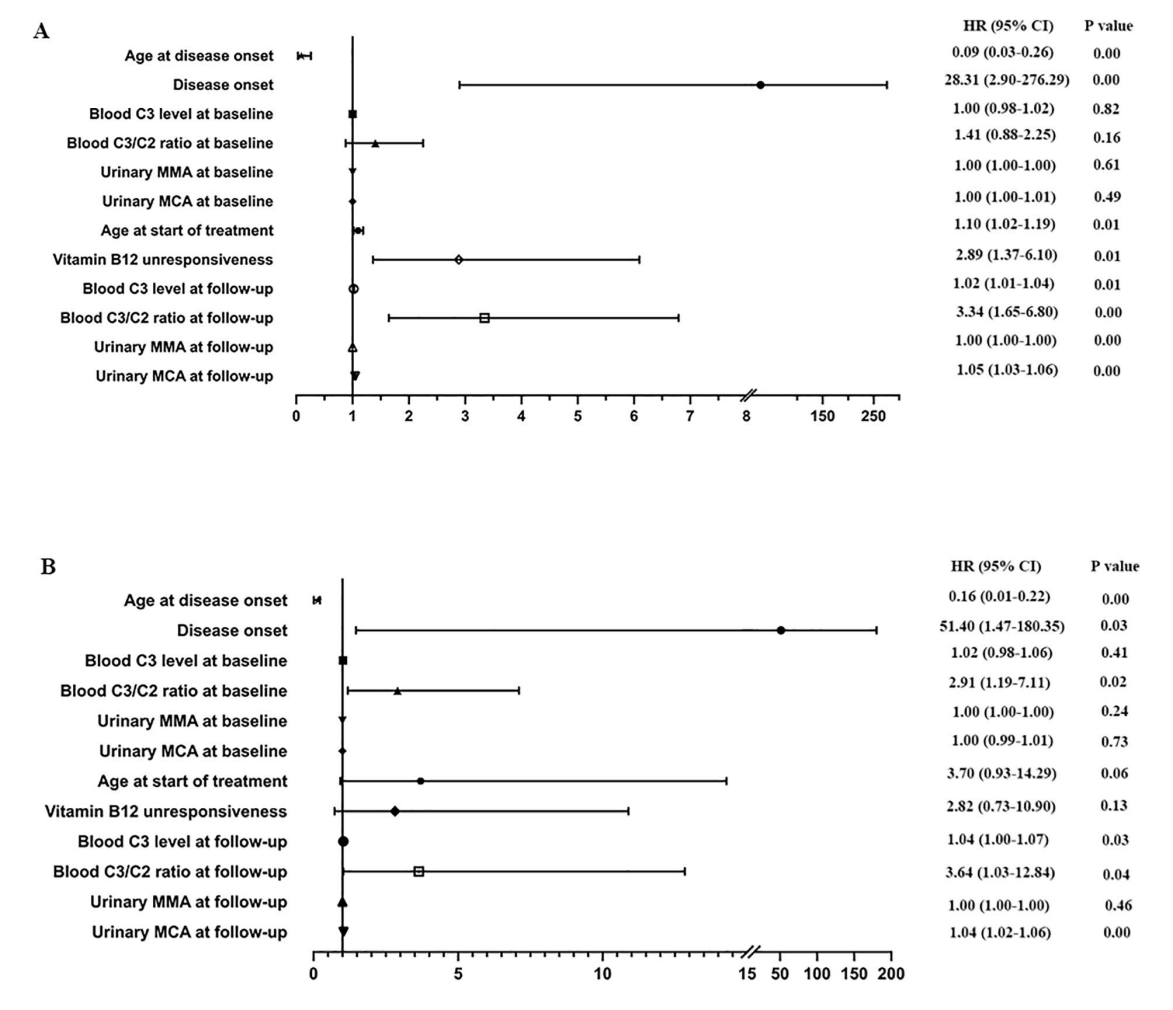
Univariate Cox regression for analyzing various predictors of morality in the whole cohort (**A**) and the NBS-detected cohort (**B**). HR: Hazard Ratio, CI: Confidence Interval

In addition, univariate Cox regression analyses were conducted for all patients, suggesting disease onset, the age at start of treatment, unresponsiveness of vitamin B12 and blood C3/C2 ratio at follow-up were positively associated with mortality. While, for patients detected by NBS, univariate Cox regression indicated disease onset, blood C3/C2 ratio at baseline and follow-up were significantly positively associated with mortality. Conversely, the age at disease onset was negatively associated with mortality no matter the patients were identified by NBS (Fig. 2).

## Discussion

Previous NBS programs for inherited metabolic diseases such as PKU greatly improved the health conditions of affected children [25, 26], facilitating the development of NBS disease panels. From this experience, it seems that overall MS/MS-based NBS has substantially improved outcomes of various metabolic disorders in general, but the long-term clinical benefits of NBS to screen infants with *mut*-type methylmalonic acidemia remain unclear. Furthermore, few studies investigated risk and prognostic factors for patients with *mut*-type identified by NBS [8, 14, 15]. Therefore, the present study evaluated long-term outcomes of patients with *mut*-type methylmalonic acidemia identified by NBS and investigated risk factors and prognostic factors with a large sample size.

In line with the previous studies [26, 27], the number of neonatally symptomatic patients increases with every day of life and the severity of neonatal metabolic decompensation is a strong predictor of impaired physical and neurocognitive development. This emphasized the need for timely diagnosis during NBS process so that the results of NBS are available earlier to avoid severe metabolic impairment which results in long-term complications or death. This study clearly demonstrated that NBS shortened the time to diagnosis for *mut*-type methylmalonic acidemia, especially for individuals with favorable clinical and cognitive long-term outcomes. Major arguments against the inclusion of *mut*-type methylmalonic acidemia in the NBS program are that C3 with a low specificity should not be considered as a screening marker for this disease and the early onset of symptoms. Indeed, in our study, a portion of patients who remained asymptomatic had normal levels of C3, while the blood C3/C2 ratio concentration was beyond the normal range. Additionally, most of these patients had high levels of MMA in urine and also carried at least one likely pathogenic mutation, thus being deemed as affected. Therefore, it’d better determine an individual’s specific diagnosis by the detection of the blood C3 level and the C3/C2 ratio combined with urinary MMA and MCA on NBS.

Compared to clinically-diagnosed individuals, our study demonstrated only a minority of screened individuals presented with clinical symptoms at a high risk of neonatal metabolic acidosis and death. Despite metabolic crises, the overall health outcome remained favorable in screened individuals, confirmed by the high survival rate (87.5%), and the less occurrence of persisting clinical signs (16.1%). In this study, the incidence of disease onset was found as one of the important risk factors for prognosis. And the overall survival was significantly better if the age at disease onset was older. Interestingly, it has become evident that the odds ratio of disease onset decreased if NBS is included, suggesting NBS could prevent neonatal metabolic crisis in about half of the screened individuals.

In accordance with the previous studies [28–30], patients in the NBS-detected cohort are more likely to have a stable clinical course with less frequent recurrences of metabolic decompensation in comparison with those diagnosed clinically. Consistent with earlier reports [7, 31, 32], milk refusal, vomiting, and drowsiness were our patients’ three most common manifestations of the initial metabolic crisis. Severe neurological manifestations such as seizures and coma tended to be more frequent in the clinically-diagnosed cohort than in the NBS-detected cohort. Similar observations have been reported that patients detected by NBS usually had symptoms at diagnosis being less severe [8]. Hyperammonemia and hyperglycemia are concomitant with acute life-threatening metabolic crises, which suggests a poor neurological prognosis [33, 34]. For the NBS-detected cohort, since early treatment reduces the accumulation of metabolites and promotes the elimination of toxic products, the incidence of hyperammonemia and hyperglycemia is decreased. In addition, chronic renal failure is one of the most important long-term complications of *mut*-type methylmalonic acidemia, and our study found that 27 patients developed chronic kidney disease, which is consistent with previous reports in the literature [7]. Furthermore, the incidence of chronic renal failure was lower in the NBS-cohort corresponding to a lower level of MMA in urine, since concentrations of methylmalonic acid are considered to be an important predictor of chronic renal failure [35, 36]. Previous investigations have focused on the direct toxicity of MMA to the liver and kidney, however, there is growing evidence that MMA can have direct toxic effects on neural tissue through overproduction of reactive oxidative species (ROS) and dysregulation of cytoskeletal elements [37, 38]. Hyperammonemia, a deadly complication of methylmalonic acidemia, is considered to be indirect toxicity and secondary effects. Therefore, most therapies have been targeted toward reducing metabolite load, centered on the toxic acylic acid metabolites [34]. Moreover, follow-up in this study confirmed that clinically-diagnosed patients had poorer outcomes than those identified by NBS. In addition, in the normal outcome group, a majority of patients remained asymptomatic, which illustrated that a later metabolic decompensation could be avoidable by early diagnosis. Therefore, a slightly earlier diagnosis by NBS is related to a milder clinical course and better outcome.

In addition, it is widely acknowledged that *mut*-type methylmalonic acidemia could be divided into two subcategories (mut^0^and mut^−^) [39], due to the absence (mut^0^) or presence (mut^−^) of residual enzyme activity in the patient’s fibroblasts by the PI assay to supplementation with hydrocobalamin [3]. Almost all mut^−^ patients usually have a better response to treatment of vitamin B12 [2, 39, 40]. Patients with *mut*-type methylmalonic acidemia usually receive the therapy combined with a low protein diet, hydrocobalamin supplementation (for responsive patients), and other oral medications in order to reduce the accumulation of toxic metabolites that eventually lead to metabolic decompensations and long-term complications [10]. In the present cohort, there is a significant interaction between cobalamin responsiveness and enzymatic subgroup on the probability of a poor outcome, with vitamin B12 unresponsiveness being the important risk factor of poor outcome. And patients had a significantly higher mortality risk if they started the treatment at an old age [15]. Therefore, vitamin B12 loading tests to evaluate the response should be applied in every *mut*-type methylmalonic acidemia patient, and for responders, vitamin B12 should be recommended as a long-term treatment.

The limitation of this study is that the follow-up information was not detailed and comprehensive enough to be recorded electronically for each patient, which is not conducive to the use of comparative statistics to accurately assess the impact of disease-specific effects, disease severity and first symptoms on clinical outcomes. Also, the median follow-up of 5 years provides limited insight into the long-term benefits of screening patients with this disease. Future electronic and systematic documentation of individual medical records in this study will hopefully fill this knowledge gap.

## Conclusion

This study illustrated that it is highly successful in applying the MS/MS-based NBS for *mut*-type methylmalonic acidemia, promptly allowing an early diagnosis and specialized metabolic therapy. NBS, allowing for early diagnosis and timely initiation of treatment, is beneficial for a favorable long-term outcome. This is confirmed by low frequencies of cognitive disabilities and premature mortalities in screened children. Therefore, NBS can improve clinical outcomes and survival and prevent disease manifestation among almost half of the screened individuals, but the onset of the disease is still the strongest factor for poor outcomes, there is a need for a safer and more effective treatment strategy for future research.

### Electronic supplementary material

Below is the link to the electronic supplementary material.

Supplementary Material 1

## Data Availability

No datasets were generated or analysed during the current study.
