# Supplementary material for: Clinical outcomes of patients with mut-type methylmalonic acidemia identified through expanded newborn screening in China
Source: Hum Genomics. 2024 Jul 29;18:84. doi: 10.1186/s40246-024-00646-0 (PMC11288086; doi:10.1186/s40246-024-00646-0)
Supplement: Supplementary file 1 — Supplementary Material 1 [file 40246_2024_646_MOESM1_ESM.docx]

Supplementary Table 1 Clinical characteristics and the mutations in the *MMUT* gene of mut-type MMA patients identified by NBS

| Case no. | Disease onset | Blood C3 level at NBS (μmol/L) | Blood C3/C2 ratio at NBS | Mutation 1 | ACMG pathogenicity | Mutation 2 | ACMG pathogenicity | Outcome at follow-up |
| --- | --- | --- | --- | --- | --- | --- | --- | --- |
| C1 | Yes | 26.86 | 1.51 | c.1630_1631GG>TA  p.G544X | LP | c.1943G>A  p.G648D | LP | Healthy |
| C2 | Yes | 16.10 | 1.25 | c.1630_1631GG>TA  p.G544X | LP | c.1943G>A  p.G648D | LP | Healthy |
| C3^a^ | Yes | 3.25 | 1.15 | c.754-1G>C  splicing | LP | c.1061C>T  p.S354F | LP | Developmental delay |
| C4 | No | 7.92 | 0.69 | c.729_730insTT  p.D244Lfs*39 | P | c.1663G>A  p.A555T | LP | Healthy |
| C5 | Yes | 14.12 | 0.71 | c.482G>T^b^  p.G161V | LP | c.482G>T^b^  p.G161V | LP | Deceased |
| C6 | Yes | 21.65 | 0.93 | c.755dupA  p.H252Qfs*6 | LP | c.1741C>T  p.R581X | P | Developmental delay |
| C7 | Yes | 8.06 | 0.64 | c.925T>G  p.W309G | LP | c.2107G>A  p.G703R | LP | Developmental delay |
| C8 | No | 5.83 | 0.31 | c.2011A>G  p.I671V | B | c.1595G>A  p.R532H | B | Healthy |
| C9 | Yes | 7.70 | 0.37 | c.914T>C  p.L305S | LP | c.1687G>C^b^  p.G563R | VUS | Lost to follow-up |
| C10 | No | 7.14 | 0.49 | c.599T>C  p.I200T | LP | c.599T>C  p.I200T | LP | Healthy |
| C11 | Yes | 5.93 | 0.60 | c.683G>A  p.R228Q | LP | c.1531C>T  p.R511X | P | Developmental delay |
| C12 | Yes | 17.39 | 0.54 | c.693C>A  p.Y231X | P | c.1159A>C  p.T387P | P | Lost to follow-up |
| C13 | Yes | 10.93 | 2.10 | c.729_730insTT  p.D244Lfs*39 | P | c.1106G>A  p.R369H | P | Deceased |
| C14 | No | 7.42 | 0.55 | c.1677-1G>A  splicing | LP | c.1943G>A  p.G648D | LP | Healthy |
| C15 | No | 6.06 | 0.31 | c.729_730insTT  p.D244Lfs*39 | P | c.1663G>A  p.A555T | LP | Healthy |
| C16 | Yes | 18.84 | 0.77 | c.323G>A  p.R108H | LP | c.494A>G  p.D165G | LP | Lost to follow-up |
| C17 | Yes | 18.20 | 0.63 | c.1677-1G>A splicing | P | c.2179C>T  p.R727X | P | Deceased |
| C18 | Yes | - | - | c.1106G>A  p.R369H | P | E12 deletion^b^ | LP | Deceased |
| C19 | Yes | 27.10 | 1.22 | c.1106G>A  p.R369H | P | c.1241_1249delAAGAATCTG^b^  p.E414_S416del | VUS | Developmental delay |
| C20 | Yes | 23.42 | 1.02 | c.467A>T  p.D156V | LP | c.729_730insTT  p.D244Lfs*39 | P | Developmental delay |
| C21 | No | 7.11 | 0.72 | c.1106G>A  p.R369H | P | c.1663G>A  p.A555T | LP | Healthy |
| C22 | No | 4.13 | 0.69 | c.755dupA  p.H252Qfs*6 | LP | c.1663G>A  p.A555T | LP | Healthy |
| C23^a^ | No | 7.14 | 0.57 | c.419T>C  p.L140p | LP | c.556A>G  p.M186V | LP | Healthy |
| C24^a^ | Yes | 4.84 | 0.18 | c.360dupT  p.K121X | P | c.1349A>G^b^  p.E450G | VUS | Healthy |
| C25 | Yes | 9.72 | 0.51 | c.424A>G  p.T142A | LP | c.1663G>A  p.A555T | LP | Developmental delay |
| C26 | Yes | 48.50 | 0.56 | c.729_730insTT  p.D244Lfs*39 | P | c.865A>G^b^  p.R289G | VUS | Developmental delay |
| C27 | No | 4.24 | 0.34 | c.556A>G  p.M186V | LP | c.1208G>A  p.R403Q | VUS | Healthy |
| C28 | No | 7.45 | 0.90 | c.103C>T  p.Q35X | P | c.755dupA  p.H252Qfs*6 | LP | Deceased |
| C29^a^ | No | 3.18 | 0.25 | c.914T>C  p.L305S | LP | c.2206C>T  p.L736F | LP | Healthy |
| C30 | Yes | 14.00 | 0.71 | c.260G>A  p.G87E | LP | c.433G>A  p.G145S | LP | Developmental delay |
| C31 | No | 5.62 | 0.57 | c.599T>C  p.I200T | LP | c.755dupA  p.H252Qfs*6 | LP | Healthy |
| C32^a^ | No | 2.15 | 0.37 | c.323G>A  p.R108H | LP | c.753+3A>G  splicing | VUS | Healthy |
| C33 | No | 15.45 | 0.22 | c.1233_1235delCAT  p.I410- | LP | c.2080C>T  p.R694W | P | Healthy |
| C34 | No | 4.53 | 0.32 | c.323G>A  p.R108H | LP | c.441T>A  p.D147E | LP | Healthy |
| C35 | No | 4.86 | 0.37 | c.424A>G  p.T142A | LP | c.1663G>A  p.A555T | LP | Healthy |
| C36 | No | 5.02 | 0.38 | c.1663G>A  p.A555T | LP | c.2131G>T  p.E711X | LP | Healthy |
| C37 | Yes | 6.20 | 0.32 | c.446A>G  p.D149G | LP | c.729_730insTT  p.D244Lfs*39 | P | Developmental delay |
| C38 | Yes | - | - | c.914T>C  p.L305S | LP | c.1630_1631insTA  p.G544Vfs*27 | P | Deceased |
| C39 | No | 4.89 | 0.33 | c.729_730insTT  p.D244Lfs*39 | P | c.1663G>A  p.A555T | LP | Healthy |
| C40 | Yes | 14.15 | 1.85 | c.914T>C  p.L305S | LP | c.1106G>A  p.R369H | P | Developmental delay |
| C41 | Yes | 9.78 | 1.06 | c.91C>T  p.R31X | P | c.729_730insTT  p.D244Lfs*39 | P | Healthy |
| C42 | No | 8.92 | 0.43 | C.1741C>T  p.R581X | P | c.2080C>T  p.R694W | P | Healthy |
| C43 | No | 8.63 | 0.39 | c.1106G>A  p.R369H | P | c.1439A>G^b^  p.D480G | VUS | Developmental delay |
| C44 | Yes | 16.85 | 0.67 | c.323G>A  p.R108H | LP | c.729_730insTT  p.D244Lfs*39 | P | Developmental delay |
| C45 | No | 6.59 | 0.24 | c.295A>C^b^  p.M99L | VUS | c.626dupC  p.K210X | P | Healthy |
| C46 | Yes | 16.20 | 1.72 | c.346G>A  p.V116M | VUS | c.1677-1G>A  splicing | P | Deceased |
| C47 | Yes | 10.87 | 0.66 | c.613G>A  p.E205K | LP | c.982C>T  p.L328F | LP | Deceased |
| C48 | Yes | 12.11 | 0.69 | c.441T>A  p.D147E | LP | c.441T>A  p.D147E | LP | Developmental delay |
| C49 | No | 9.46 | 0.82 | c.544dupA  p.M182Nfs*29 | P | c.1106G>A  p.R369H | P | Lost to follow-up |
| C50^a^ | No | 7.57 | 0.17 | c.1880A>G  p.H627R | LP | c.1919A>T  p.D640V | VUS | Healthy |
| C51 | Yes | 14.48 | 0.65 | c.323G>A  p.R108H | LP | c.424A>G  p.T142A | LP | Lost to follow-up |
| C52 | Yes | 13.50 | 1.53 | c.914T>C  p.L305S | LP | c.2062G>T  p.E688X | P | Developmental delay |
| C53 | No | 6.67 | 0.69 | c.1106G>A  p.R369H | P | c.1663G>A  p.A555T | LP | Healthy |
| C54 | Yes | 10.05 | 0.42 | c.729_730insTT  p.D244Lfs*39 | P | c.1956+1delG  splicing | LP | Deceased |
| C55 | Yes | 7.86 | 1.04 | c.1106G>A  p.R369H | P | c.1741C>T  p.R581X | P | Developmental delay |
| C56 | Yes | 5.83 | 0.60 | c.1280G>A  p.G427D | LP | c.1677-1G>A  splicing | LP | Deceased |
| C57 | Yes | 50.74 | 1.94 | C.1677-1G>A  splicing | LP | c.2156delC^b^  p.N720fs | LP | Deceased |
| C58 | Yes | 4.91 | 0.75 | c.454C>T  p.R152X | P | c.2080C>T  P.R694W | P | Healthy |
| C59 | Yes | 9.91 | 1.42 | c.729_730insTT  p.D244Lfs*39 | P | c.2080C>T  P.R694W | P | Developmental delay |
| C60 | No | 14.05 | 1.02 | c.323G>A  p.R108H | LP | c.424A>G  p.T142A | LP | Healthy |
| C61 | Yes | 5.55 | 0.30 | c.323G>A  p.R108H | LP | c.914T>C  p.L305S | LP | Developmental delay |
| C62 | No | 5.05 | 0.31 | c.753+3A>G  splicing | VUS | c.755dupA  p.H252Qfs*6 | LP | Healthy |
| C63 | Yes | 10.32 | 0.54 | c.278G>A  p.R93H | P | c.1106G>A  p.R369H | P | Developmental delay |
| C64 | Yes | - | - | c.424A>G  p.T142A | LP | c.1159A>C  p.T387P | P | Developmental delay |
| C65 | Yes | 9.49 | 0.48 | c.424A>G  p.T142A | LP | c.2080C>T  p.R694W | P | Developmental delay |
| C66^a^ | No | 2.16 | 0.37 | c.419T>C  p.L140p | LP | c.2131G>T  p.E711X | LP | Healthy |
| C67 | No | 13.08 | 0.67 | c.494A>G  p.D165G | LP | c.1630_1631GG>TA  p.G544X | LP | Healthy |
| C68 | Yes | 10.36 | 0.87 | c.729_730insTT  p.D244Lfs*39 | P | c.2107G>A  p.G703R | LP | Healthy |
| C69 | Yes | 9.20 | 0.60 | c.349G>T  p.E117X | P | c.1106G>A  p.R369H | P | Developmental delay |
| C70 | Yes | 5.85 | 0.39 | c.1663G>A  p.A555T | LP | c.1280G>A  p.G427D | LP | Developmental delay |
| C71 | No | - | 0.52 | c.566A>G  p.N189S | VUS | Not detected | - | Healthy |
| C72 | No | 5.82 | 0.30 | c.1663G>A  p.A555T | LP | E13 deletion^b^ | LP | Healthy |
| C73 | Yes | 21.10 | 0.79 | c.1106G>A  p.R369H | P | c.2131G>T  p.E711X | LP | Developmental delay |
| C74 | Yes | 13.61 | 0.85 | c.1280G>A  p.G427D | LP | c.1677-1G>A  splicing | LP | Lost to follow-up |
| C75 | Yes | 4.53 | 0.26 | c.599T>C  p.I200T | LP | c.1106G>A  p.R369H | P | Developmental delay |
| C76 | Yes | 9.81 | 1.08 | c.1679G>A  p.C560Y | LP | c.1850T>G  p.L617R | VUS | Developmental delay |
| C77 | No | 6.98 | 0.69 | c.729_730insTT  p.D244Lfs*39 | P | c.1663G>A  p.A555T | LP | Healthy |
| C78 | No | 5.94 | 0.45 | c.1233_1235delCAT  p.I411- | LP | c.1663G>A  p.A555T | LP | Healthy |
| C79 | Yes | 6.50 | 1.40 | c.322C>T  p.R108C | P | c.581C>T  p.P194L | LP | Developmental delay |
| C80 | No | 4.64 | 0.21 | c.1474T>A^b^  p.Y492N | VUS | Not detected | - | Healthy |
| C81 | Yes | 8.54 | 0.59 | c.729_730insTT  p.D244Lfs*39 | P | c.1679G>A  p.C560Y | LP | Deceased |
| C82 | No | 5.54 | 1.28 | c.729_730insTT  p.D244Lfs*39 | P | c.2216T>C  p.I739T | LP | Healthy |
| C83 | Yes | 7.88 | 0.69 | c.424A>G  p.T142A | LP | c.1537_1538insT^b^  p.R513Mfs*4 | LP | Developmental delay |
| C84 | Yes | 28.74 | 1.54 | c.729_730insTT  p.D244Lfs*39 | P | c.729_730insTT  p.D244Lfs*39 | P | Developmental delay |
| C85 | Yes | 7.92 | 0.90 | c.424A>G  p.T142A | LP | c.544dupA  p.M182Nfs*29 | P | Developmental delay |
| C86 | No | 10.67 | 0.29 | c.659A>T^b^  p.D220V | VUS | c.1943G>A  p.G648D | LP | Healthy |
| C87 | No | 7.68 | 0.34 | c.323G>A  p.R108H | LP | c.424A>G  p.T142A | LP | Healthy |
| C88 | Yes | 42.94 | 4.30 | c.729_730insTT  p.D244Lfs*39 | P | c.729_730insTT  p.D244Lfs*39 | P | Deceased |
| C89 | Yes | - | - | c.1106G>A  p.R369H | P | c.1677-1G>A  splicing | LP | Deceased |
| C90 | Yes | 54.85 | 0.80 | c.682C>T  p.R228X | P | c.1420C>T  p.R474X | P | Developmental delay |
| C91 | Yes | 5.10 | 0.77 | c.433G>T^b^  p.G145C | VUS | c.2179C>T  p.R727X | P | Deceased |
| C92 | Yes | 9.61 | 0.88 | c.288_289insA^b^  p.P97Tfs | LP | c.1159A>C  p.T387P | P | Deceased |
| C93^a^ | No | 2.11 | 0.22 | c.626dupC  p.K210X | P | c.1208G>A  p.R403Q | VUS | Healthy |
| C94 | Yes | 11.77 | 0.88 | c.729_730insTT  p.D244Lfs*39 | P | c.1106G>A  p.R369H | P | Developmental delay |
| C95 | Yes | 5.61 | 0.51 | c.360dupT  p.K121X | P | c.1677-1G>A  splicing | LP | Deceased |
| C96 | Yes | 8.36 | 0.57 | c.1679G>A  p.C560Y | LP | Not detected | - | Developmental delay |
| C97 | Yes | 6.70 | 1.22 | c.965T>A^b^  p.M322K | VUS | c.1630_1631GG>TA  p.G544X | LP | Developmental delay |
| C98 | Yes | 4.89 | - | c.1105C>T  p.R369C | P | c.323G>A  p.R108H | LP | Deceased |
| C99 | No | 19.09 | 1.26 | c.729_730insTT  p.D244Lfs*39 | P | c.421G>A  p.A141T | LP | Healthy |
| C100 | No | 13.00 | 0.54 | c.1630_1631GG>TA  p.G544X | LP | c.1630_1631GG>TA  p.G544X | LP | Healthy |
| C101 | No | 4.20 | 0.48 | c.424A>G  p.T142A | LP | c.753+3A>G  splicing | VUS | Healthy |
| C102 | Yes | 11.53 | 0.74 | c.1777G>T  p.E593X | P | c.729_730insTT  p.D244Lfs*39 | P | Developmental delay |
| C103 | No | 5.03 | 0.22 | c.278G>A  p.R93H | P | c.1784_1785delAA  p.K595Rfs*11 | LP | Healthy |
| C104 | Yes | 6.76 | 0.62 | c.1663G>A  p.A555T | LP | c.914T>C  p.L305S | LP | Healthy |
| C105 | No | 13.41 | 1.22 | c.2168G>A  p.G723D | LP | c.920_923delTCTT  p.F307Sfs*6 | P | Healthy |
| C106^a^ | No | 6.06 | 0.12 | c.323G>A  p.R108H | LP | c.1610T>A^b^  p. L537Q | VUS | Healthy |
| C107^a^ | No | 3.78 | 0.24 | c.788G>T  p. G263V | LP | c.724A>T^b^  p.I242F | VUS | Healthy |
| C108 | No | 4.10 | 0.34 | c.1663G>A  p.A555T | LP | c.1679G>A  p.C560Y | LP | Healthy |
| C109^a^ | No | 5.82 | 0.18 | c.1663G>A  p.A555T | LP | c.1106G>A  p.R369H | P | Healthy |
| C110 | No | 6.54 | 0.33 | c.1663G>A  p.A555T | LP | c.1207C>T  p. R403X | P | Healthy |
| C111 | Yes | - | - | c.277C>T  p.R93C | LP | c.1106G>A  p.R369H | P | Developmental delay |
| C112 | No | 2.4 | 0.32 | c.1663G>A  p.A555T | LP | c.2009G>T  p.G670V | LP | Healthy |
| C113 | No | 4.75 | 0.38 | c.556A>G  p.M186V | LP | c.1106G>A  p.R369H | P | Healthy |
| C114^a^ | No | 3.98 | 0.26 | c.494A>G  p.D165G | LP | c.1663G>A  p.A555T | LP | Healthy |
| C115 | No | 4.63 | 0.22 | c.431G>A  p.R144H  c.1286A>G  p.Y429C | LP  LP | c.1138G>A  p.G380R | VUS | Healthy |
| C116 | No | 5.63 | 0.59 | c.1847G>A^b^  p.R616H | VUS | Not detected | - | Healthy |
| C117 | Yes | 10.56 | 0.57 | c.729_730insTT  p.D244Lfs*39 | P | c.914T>C  p.L305S | LP | Deceased |
| C118 | Yes | 7.56 | 0.41 | c.914T>C  p.L305S | LP | c.1233_1235delCAT  p.I410- | LP | Deceased |
| C119 | Yes | 10.40 | 1.73 | c.1880A>G  p.H627R | LP | c.1280G>A  p.G427D | LP | Developmental delay |
| C120 | Yes | 14.583 | 0.613 | c.626dupC  p.K210X | P | c.494A>G  p.D165G | LP | Healthy |
| C121 | No | - | - | c.1663G>A  p.A555T | LP | c.729_730insTT  p.D244Lfs*39 | P | Healthy |
| C122 | No | 4.71 | 0.41 | c.1663G>A  p.A555T | LP | Not detected | - | Healthy |
| C123 | Yes | 9.3 | 0.94 | c.323G>A  p.R108H | LP | c.729_730insTT  p.D244Lfs*39 | P | Developmental delay |
| C124 | No | 7.88 | 0.49 | c.1663G>A  p.A555T | LP | c.729_730insTT  p.D244Lfs*39 | P | Healthy |
| C125 | No | 36.00 | 0.93 | c.729_730insTT  p.D244Lfs*39 | P | c.1009T>C  p.F337L | LP | Healthy |
| C126 | No | 11.79 | 0.53 | c.2080C>T  p.R694W | P | c.1105C>T  p.R369C | P | Healthy |
| C127 | No | 3.8 | 0.52 | c.1663G>A  p.A555T | LP | c.729_730insTT  p.D244Lfs*39 | P | Healthy |
| C128 | Yes | 6.45 | 0.58 | c.1673C>T^b^  p. A558V | VUS | c.2179C>T  p.R727X | P | Developmental delay |
| C129 | Yes | 10.76 | 2.06 | c.975_976delTA^b^  p.R326Kfs*14 | LP | c.914T>C  p.L305S | LP | Developmental delay |
| C130 | No | 7.41 | 0.34 | c.1142G>A^b^  p.G381E | VUS | c.1153_1154delTT  p.L385Afs*6 | LP | Healthy |
| C131 | Yes | 27.56 | 0.68 | C.1070C>G^b^  p.S357X | LP | c.268delinsAA^b^  p.P99Nfs*14 | LP | Lost to follow-up |
| C132 | Yes | 21.11 | 0.68 | c.91C>T  p.R31X | P | c.1850T>G  p.L617R | VUS | Developmental delay |
| C133 | No | 4.63 | 0.23 | c.1663G>A  p.A555T | LP | c.2131G>T  p.E711X | LP | Healthy |
| C134 | Yes | 9.94 | 0.60 | c.103C>T  p.Q35X | P | c.322C>T  p.R108C | LP | Developmental delay |
| C135 | Yes | 4.67 | 0.34 | c.1663G>A  p.A555T | LP | Not detected | - | Healthy |
| C136 | No | - | - | c.1280G>A  p.G427D | LP | c.1280G>A  p.G427D | LP | Healthy |
| C137 | Yes | 7.81 | 0.92 | c.1663G>A  p.A555T | LP | c.626dupC  p.K210X | P | Healthy |
| C138 | No | 15.58 | 0.77 | c.1677-1G>A  splicing | LP | c.729_730insTT  p.D244Lfs*39 | P | Healthy |
| C139 | Yes | 16.68 | 0.85 | c.323G>A  p.R108H | LP | c.914T>C  p.L305S | LP | Developmental delay |
| C140 | Yes | 13.97 | 0.96 | c.755dupA  p.H252Qfs*6 | LP | c.29dupT  p.L10Ffs*39 | P | Developmental delay |
| C141 | Yes | 10.32 | 0.54 | c.278G>A  p.R93H | P | c.1106G>A  p.R369H | P | Developmental delay |
| C142 | No | 13.61 | 1.06 | c.754-1G>C  splicing | P | c.2009G>T  p.G670V | LP | Healthy |
| C143 | Yes | 5.11 | 0.47 | c.599T>C  p.I200T | LP | c.1741C>T  p.R581X | P | Developmental delay |
| C144 | Yes | 9.55 | 0.84 | c.323G>A  p.R108H | LP | c.914T>C  p.L305S | LP | Healthy |
| C145 | Yes | 4.92 | 0.71 | c.755dupA  p.H252Qfs*6 | LP | c.1531C>T  p.R511X | P | Developmental delay |
| C146 | Yes | 13.59 | 0.66 | c.457G>A^b^  p.V53I | VUS | Not detected | - | Developmental delay |
| C147 | Yes | 4.95 | 0.55 | c.323G>A  p.R108H | LP | c.1106G>A  p.R369H | P | Developmental delay |
| C148 | Yes | 4.71 | 1.52 | c.729_730insTT  p.D244Lfs*39 | P | c.1445-2A>G^b^  splicing | P | Deceased |
| C149 | No | 11 | 0.51 | c.1677-1G>A  splicing | LP | c.599T>C  p.I200T | LP | Healthy |
| C150 | No | 10.71 | 1.18 | c.755dupA  p.H252Qfs*6 | LP | c.599T>C  p.I200T | LP | Healthy |
| C151 | No | 4.57 | 0.36 | c.755dupA  p.H252Qfs*6 | LP | c.599T>C  p.I200T | LP | Healthy |
| C152 | Yes | 14.00 | 0.44 | c.1106G>A  p.R369H | P | c.494A>G  p.D165G | LP | Lost to follow-up |
| C153^a^ | No | 4.55 | 0.19 | c.1663G>A  p.A555T | LP | c.1106G>A  p.R369H | P | Healthy |
| C154 | Yes | 7.88 | 0.49 | c.1663G>A  p.A555T | LP | c.729_730insTT  p.D244Lfs*39 | P | Healthy |
| C155 | No | 6.90 | 0.42 | c.1280G>A  p.G427D | LP | c.613G>A  p.E205K | LP | Developmental delay |
| C156 | No | 40.42 | 0.29 | c.729_730insTT  p.D244Lfs*39 | P | c.1663G>A  p.A555T | LP | Healthy |
| C157^a^ | No | 3.74 | 0.30 | c.626dupC  p.K210X | P | c.1663G>A  p.A555T | LP | Healthy |
| C158 | No | 10.71 | 1.18 | c.755dupA  p.H252Qfs*6 | LP | c.599T＞C  p.I200T | LP | Healthy |
| C159 | Yes | 9.77 | 0.27 | c.729_730insTT  p.D244Lfs*39 | P | c.1630G>T  p.G544X | LP | Developmental delay |
| C160 | Yes | 4.27 | 0.47 | c.1630G>T  p.G544X | LP | c.1676+11A>G  splicing | VUS | Healthy |
| C161 | Yes | 10.81 | 0.73 | c.1105C>T  p.R369C | P | c.1105C>T  p.R369C | P | Healthy |
| C162 | No | 39.94 | 1.72 | c.323G>A  p.R108H | LP | c.835T>G^b^  p.Y279D | VUS | Healthy |
| C163 | No | 8.29 | 0.51 | c.729_730insTT  p.D244Lfs*39 | P | c.1663G>A  p.A555T | LP | Healthy |
| C164^a^ | No | 2.35 | 0.33 | c.433G>A  p.G145S | LP | c.1663G>A  p.A555T | LP | Healthy |
| C165 | No | 7.55 | 0.31 | c.729_730insTT  p.D244Lfs*39 | P | c.1663G>A  p.A555T | LP | Healthy |
| C166 | Yes | 48.09 | 3.55 | c.729_730insTT  p.D244Lfs*39 | P | c.1106G>A  p.R369H | LP | Developmental delay |
| C167 | No | 4.19 | 0.27 | c.323G>A  p.R108H | LP | c.1663G>A  p.A555T | LP | Healthy |
| C168 | No | 5.18 | 0.46 | c.1663G>A  p.A555T | LP | c.1106G>A  p.R369H | P | Healthy |

Normal reference range of blood C3: 0.5-4.0μmol/L; Normal reference range of blood C3/C2 ratio: 0.04-0.2

Abbreviation: P pathogenic, LP likely pathogenic, VUS uncertain significance, B benign

^a^Patients either with the normal blood C3 level or C3/C2 ratio at NBS

^b^Mutations have not been reported in HGMD database, ClinVar database, and previous literatures

-, not available
